# Stretch of the papillary insertion triggers reentrant arrhythmia: an *in silico* patient study

**DOI:** 10.3389/fphys.2024.1447938

**Published:** 2024-08-19

**Authors:** Lena Myklebust, Giulia Monopoli, Gabriel Balaban, Eivind Westrum Aabel, Margareth Ribe, Anna Isotta Castrini, Nina Eide Hasselberg, Cecilie Bugge, Christian Five, Kristina Haugaa, Mary M. Maleckar, Hermenegild Arevalo

**Affiliations:** ^1^ Computational Physiology Department, Simula Research Laboratory, Oslo, Norway; ^2^ School of Economics Innovation and Technology, Kristiania University College, Oslo, Norway; ^3^ ProCardio Center for Innovation, Department of Cardiology, Oslo University Hospital, Oslo, Norway; ^4^ Institute of Clinical Medicine, Faculty of Medicine, University of Oslo, Oslo, Norway

**Keywords:** mitral annulus disjunction, mitral valve prolapse, ventricular reentry, stretch activated channels, computational modeling, cardiac electrophysiology, stretch induced arrhythmia, mechanical stretch

## Abstract

**Background:**

The electrophysiological mechanism connecting mitral valve prolapse (MVP), premature ventricular complexes and life-threatening ventricular arrhythmia is unknown. A common hypothesis is that stretch activated channels (SACs) play a significant role. SACs can trigger depolarizations or shorten repolarization times in response to myocardial stretch. Through these mechanisms, pathological traction of the papillary muscle (PM), as has been observed in patients with MVP, may induce irregular electrical activity and result in reentrant arrhythmia.

**Methods:**

Based on a patient with MVP and mitral annulus disjunction, we modeled the effect of excessive PM traction in a detailed medical image-derived ventricular model by activating SACs in the PM insertion region. By systematically varying the onset of SAC activation following sinus pacing, we identified vulnerability windows for reentry with 1 ms resolution. We explored how reentry was affected by the SAC reversal potential $\left(E_{\text{SAC}}\right)$ and the size of the region with simulated stretch (SAC region). Finally, the effect of global or focal fibrosis, modeled as reduction in tissue conductivity or mesh splitting (fibrotic microstructure), was investigated.

**Results:**

In models with healthy tissue or fibrosis modeled solely as CV slowing, we observed two vulnerable periods of reentry: For $E_{\text{SAC}}$ of −10 and −30 mV, SAC activated during the T-wave could cause depolarization of the SAC region which lead to reentry. For $E_{\text{SAC}}$ of −40 and −70 mV, SAC activated during the QRS complex could result in early repolarization of the SAC region and subsequent reentry. In models with fibrotic microstructure in the SAC region, we observed micro-reentries and a larger variability in which times of SAC activation triggered reentry. In these models, 86% of reentries were triggered during the QRS complex or T-wave. We only observed reentry for sufficiently large SAC regions ( >= 8 mm radius in models with healthy tissue).

**Conclusion:**

Stretch of the PM insertion region following sinus activation may initiate ventricular reentry in patients with MVP, with or without fibrosis. Depending on the SAC reversal potential and timing of stretch, reentry may be triggered by ectopy due to SAC-induced depolarizations or by early repolarization within the SAC region.

## 1 Introduction

Mitral valve prolapse (MVP), a displacement of a part of the mitral valve into the left atrium, is a common abnormality in the general population, with a reported prevalence of about 2.4% (Freed et al., 1999). Associated with MVP is mitral annulus disjunction (MAD), a separation between the ventricular myocardium and the mitral annulus (Moscatelli and Antonakaki, 2023). Although MVP with or without MAD generally has a good prognosis, a small subset of patients experience life-threatening ventricular arrhythmias (Nishimura et al., 1985; Miller et al., 2018). The electrophysiological mechanism of arrhythmia in these patients is still unclear, and various explanations have been proposed. A leading hypothesis is that arrhythmia may be initiated by premature ventricular complexes (PVCs) triggered by excessive stretch of the papillary muscles (PMs) and inferobasal wall by prolapsing mitral leaflets (Basso and Perazzolo Marra, 2018; Enriquez et al., 2019; Hei et al., 2019). Suggesting the presence of such stretch, studies on MVP patients have reported abnormal myocardial strain patterns (Huttin et al., 2017; Nagata et al., 2023), an abnormal shift of the PMs towards the mitral annulus (Sanfilippo et al., 1992; Hei et al., 2019) as well as fibrosis, inflammation or ischemia within the PMs (Dejgaard et al., 2018; Fulton et al., 2018; Miller et al., 2020; Figliozzi et al., 2023).

It is widely recognized that myocardial stretch can lead to local changes in the membrane potential *via* activation of stretch activated channels (SACs) (Reed et al., 2014; Quinn and Kohl, 2021). In a myocardial cell, depending on the reversal potential of SAC $\left(E_{\text{SAC}}\right)$ , and timing with respect to the previous action potential (AP), the resulting effect can be either depolarizing or repolarizing (Zabel et al., 1996). This in turn may elicit PVCs and/or cause repolarization abnormalities leading to arrhythmia. While the latter mechanism is still unclear, stretch-induced depolarizations as an arrhythmic mechanism has been well documented (Stacy Jr et al., 1992; Dick and Lab, 1998; Quinn et al., 2017). Specifically relating PM stretch to PVCs, mechanical PM traction has been shown to cause early activations in the cardiac ventricle of dogs (Gornick et al., 1986). Manual traction of chordae tendineae attached to the PM has also been shown to reproduce spontaneous PVCs originating near the PM in an MVP patient (Wilde et al., 1997). Furthermore, recent clinical studies propose that mechanical traction of the PMs can trigger PVCs or arrhythmia in patients with MVP (Basso et al., 2019). Finally, clinical work suggest an association between frequent PVCs and severe arrhythmia in patients with MVP and/or MAD (Aabel et al., 2023), and PVCs originating from the PMs have been shown to trigger ventricular fibrillation in these patients (Enriquez et al., 2019).

While the above-mentioned studies have addressed different components to the relation between MVP/MAD, stretch and arrhythmia, the electrophysiological mechanism of how PM stretch may cause sustained arrhythmia is still an open question. Computational electrophysiological studies have investigated stretch-induced reentry in 2D (Garny and Kohl, 2004), in 3D simulations of commotio cordis following electrical pacing from the apex (Li et al., 2004) and within a region of acute ischemia in the rabbit ventricle (Jie et al., 2010). However, the conditions required to trigger ectopy and subsequent reentry from stretch of the PM region following sinus rhythm, potentially linked to MVP and MAD, remains unexplored.

In this study, we simulated sinus activity followed by SAC activation in the PM insertion region of the human cardiac ventricles. Simulations were conducted in an electrophysiological model based on cardiovascular magnetic resonance (CMR) images of a patient with MVP and concomitant MAD. We thus demonstrated that depending on $E_{\text{SAC}}$ , activation of SAC during the QRS complex or the T-wave can induce reentrant arrhythmia. By varying $E_{\text{SAC}}$ , timing of stretch and size of the SAC region we investigated conditions for reentry. Additionally, motivated by evidence of global or PM-related fibrosis in these patients (Dejgaard et al., 2018; Fulton et al., 2018; Chivulescu et al., 2022; Figliozzi et al., 2023), we explored the effect of conduction slowing and fibrotic microstructure on reentry inducibility and sustainability.

## 2 Materials and methods

### 2.1 Image data and geometrical mesh construction

To construct the ventricular geometry, we used short-axis late gadolinium-enhanced CMR images of a single patient with MVP and concomitant MAD, provided by the Oslo University Hospital (Aabel et al., 2021). The patient presented with PM fibrosis, PVCs and sustained ventricular tachycardia. The images consisted of 14 slices, with a resolution of 0.91 mm and slice thickness of 10 mm. Using the software Segment (Heiberg et al., 2010), we manually identified the epi- and endocardium of both ventricles. An openly available automatic workflow (Marciniak et al., 2016; Marciniak, 2017) was used to construct a 3D finite element model of the segmented ventricle. The pipeline involves initial slice alignment and the subsequent construction of surfaces using the Visualization Toolkit (Schroeder et al., 1998). From these surfaces, a finite element ventricular model was created using gmsh (Geuzaine and Remacle, 2009). The final model consisted of 6,173,699 elements with a mean resolution of 530 μ m. Using the software by Finsberg (2022), we assigned myocardial fiber orientations according to the Laplace-Dirichlet Rule-Based (LDRB) algorithm (Bayer et al., 2012).

### 2.2 Spatial SAC region

**FIGURE 1:**
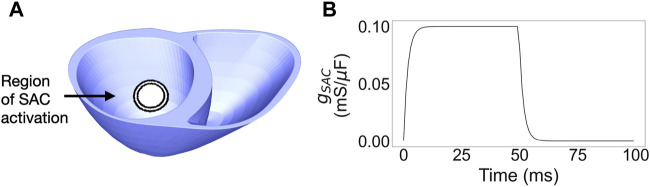
**(A)** Ventricular mesh with the layered SAC region, from 7 to 10 mm radius. **(B)** Time dependence of the SAC conductance, $g_{\text{SAC}}$ . SAC: Stretch activated channels.

We represented the effect of mechanical traction on the PM by activating SAC currents in a specific region of tissue, from here on referred to as the SAC region. Since PVCs originating from either of the PMs have been reported in multiple MVP patients (Wilde et al., 1997; Fulton et al., 2018; Conti et al., 2023), we selected a point representative of the inferior PM insertion point for our SAC region. Specifically, the SAC region (Figure 1A) was defined as a spherical region centered in a point in the inferior left ventricular wall, 1/3 of the apico-basal distance away from the apex (Carpentier et al., 2010).

Large individual variations exist regarding both PM shape and PM attachment to the ventricular wall (Rajiah et al., 2019). The latter may range from a single point of attachment to an extensive network of trabeculae connected to varying portions of the wall (Ranganathan and Burch, 1969; Axel, 2004). To investigate how the size of the stretched region affected reentry, we created models with varying radii of the SAC region. Starting from 10 mm, we reduced the radius in steps of 1 mm to investigate which size of the SAC region was needed to induce reentry. If we could no longer induce reentry after shrinking the SAC region, we did not reduce it further. Thus the smallest region used for simulations was of 7 mm radius. For an overview of all ventricular models and their SAC regions, see Table 1.

**TABLE 1:** Parameter combinations for models included in the main manuscript. For all listed combinations, we activated SAC for a duration of 50 ms, with a maximum conductance $g_{\text{SACtarget}}$ = 0.1 mS/ μ F. The first column indicates the name used for referring to a type of model. Radius refers to the area with SAC activation. The models ${M1}_{9}$ - ${M7}_{9}$ , ${M1}_{8}$ - ${M7}_{8}$ and ${M7}_{7}$ have SAC radii of 9, 8 and 7 mm, respectively, while models M1-M7 without subscript have SAC radii of 10 mm. Since the models M1-M6 were non-inducible for 8 mm SAC radius, we did not further reduce the SAC radius in these models. Subscripts for models M8-M14 represent the degree (20, 40 or 60%) and type of conduction slowing (g = global and l = local slowing). Δ APDs describes the global APD heterogeneity by referring to the minimum and maximum APD in the tissue, resulting from the selected set of $I_{\text{Ks}}$ scaling factors in the model. BCL refers to the duration between each sinus pacing. Columns with FM refers to models with fibrotic microstructure in the SAC region. Columns with CVS refers to models with CV slowing. $E_{\text{SAC}}$ : resting potential of the SAC current (mV). CV: Tissue conduction velocity. BCL: basic cycle length.

Name	Radius (mm)	$E_{SAC}$ (mV)	Tissue type	BCL (ms)	Δ APDs (ms)
M1, ${M1}_{9/8}$	10, 9, 8	−10	Healthy	500	206:290
M2, ${M2}_{9/8}$	10, 9, 8	−20	Healthy	500	206:290
M3, ${M3}_{9/8}$	10, 9, 8	−30	Healthy	500	206:290
M4, ${M4}_{9/8}$	10, 9, 8	−40	Healthy	500	206:290
M5, ${M5}_{9/8}$	10, 9, 8	−50	Healthy	500	206:290
M6, ${M6}_{9/8}$	10, 9, 8	−60	Healthy	500	206:290
M7, ${M7}_{9/8/7}$	10, 9, 8, 7	−70	Healthy	500	206:290
${M8}_{20/40/60,g/l}$	10	−10	CVS	500	206:290
${M9}_{20/40/60,g/l}$	10	−20	CVS	500	206:290
${M10}_{20/40/60,g/l}$	10	−30	CVS	500	206:290
${M11}_{20/40/60,g/l}$	10	−40	CVS	500	206:290
${M12}_{20/40/60,g/l}$	10	−50	CVS	500	206:290
${M13}_{20/40/60,g/l}$	10	−60	CVS	500	206:290
${M14}_{20/40/60,g/l}$	10	−70	CVS	500	206:290
M15	10	−10	FM	500	206:290
M16	10	−20	FM	500	206:290
M17	10	−30	FM	500	206:290
M18	10	−40	FM	500	206:290
M19	10	−50	FM	500	206:290
M20	10	−60	FM	500	206:290
M21	10	−70	FM	500	206:290

### 2.3 Electrophysiology

#### 2.3.1 Electrophysiological models

Tissue propagation was represented with the monodomain model as given by:

$$\nabla \cdot \left(\sigma \nabla V_{\text{m}}\right)=\chi \left(C_{\text{m}}\frac{\partial V_{\text{m}}}{\partial t}+I_{\text{ion}}\right),$$ (1)

where $V_{\text{m}}$ is the membrane potential, t is the time, χ is the surface-to-volume ratio of the membrane, $C_{\text{m}}$ is the membrane capacitance per unit area and $I_{\text{ion}}$ is the membrane ionic currents density (Keener and Sneyd, 1998). The parameter σ is a conductivity tensor given by:

$$\sigma =\sigma_{\text{i}}\sigma_{\text{e}}{\left(\sigma_{\text{i}}+\sigma_{\text{e}}\right)}^{-1},$$ (2)

where $\sigma_{\text{i}}$ and $\sigma_{\text{e}}$ are the intracellular and extracellular conductivity tensors, respectively (Keener and Sneyd, 1998). We used the ten Tusscher model of a healthy human ventricular cardiomyocyte to represent the membrane kinetics (Ten Tusscher and Panfilov, 2006).

#### 2.3.2 Action potential heterogeneity

Various types of heterogeneity in action potential duration (APD), including patient-specific variations, have been reported clinically (Opthof et al., 2017). In our model, in accordance with previous work by Sung et al. (2021) and Keller et al. (2011), we applied linear APD gradients along the apicobasal and transmural direction by scaling the slow delayed rectifier K+ current $\left(I_{\text{Ks}}\right)$ . For the apico-basal direction, we defined the gradient based on the apico-basal axis of our mesh. Transmurally, the gradient was defined by computing the Laplace solution between the endo- and epicardium, as done previously (Bayer et al., 2018; Sung et al., 2021). Compared to the scaling factor used in Sung et al. (2021) and Keller et al. (2011) (1-1.5 in both directions, resulting in an APD gradient of 241–290 ms in our model) we used a higher scaling factor (1-2 in both directions). This scaling factor was chosen to achieve a wider APD gradient, more in line with differences in activation-recovery intervals of ∼ 100 ms reported in recent clinical measurements (Opthof et al., 2017). This resulted in total scaling factors from one in the basal endocardial cell to four in the apical epicardial cell, with maximum and minimum APDs of 290 and 206 ms, respectively.

We refer to the APD heterogeneity in this study by noting the minimum and maximum APDs when measured at 90 % repolarization: ${APD}_{\mathrm{206-290}}$ . In addition to the gradient described above, we ran test simulations for two other APD gradients to check how this affected reentry formation. In the first ( ${APD}_{\mathrm{241-290}}$ ), we used the original scaling factors reported by Sung et al. (2021) and Keller et al. (2011). In the second ( ${APD}_{\mathrm{188-235}}$ ), we applied an overall shortening of APDs. The methods and results for the two additional gradients are reported in the Supplementary Material (Section 1.2; Supplementary Figure S2). The APD gradients used in each ventricular model are given in Table 1 and in Supplementary Table S2 in Supplementary Material.

#### 2.3.3 Conductivities

We considered three types of tissue conductivity properties in our models: one with only healthy tissue, one with diffuse fibrosis and one with fibrosis in the SAC region. In the healthy case we tuned the model tissue conductivities (given by σ in Equations 1 and 2 to achieve longitudinal and transverse velocities of 0.56 and 0.21 m/s, respectively, according to clinical measurements of normal ventricular conduction (Kléber et al., 2011).

For modelling fibrosis in models ${M8-M14}_{20/40/60,g/l}$ (Table 1) we applied reductions to the tissue conductivities. To represent diffuse fibrosis ( ${M8-M14}_{20/40/60,g}$ , Table 1) we reduced the conductivity in the entire myocardium. Such fibrosis has been reported in MVP patients using T1 mapping (Bui et al., 2017) and could influence arrhythmogenesis. In models with fibrosis in the SAC region ( ${M8-M14}_{20/40/60,l}$ we reduced the conductivity in the SAC region only, representing focal fibrosis. In patients with MVP and/or MAD, fibrosis close to or within the PMs have been linked to arrhythmia (Dejgaard et al., 2018; Nagata et al., 2023).

A wide range of CV values have been reported in fibrotic tissue, depending on fibrosis type and severity (Anderson et al., 1993; Kawara et al., 2001). Thus, for both types of fibrosis (diffuse or focal), we created three models with different levels of conduction slowing: 20%, 40% and 60% CV reduction. All CVs were tuned using a 10 cm rod of myocardial tissue with 530 μ m resolution, equal to the mean resolution of the ventricular mesh. The tuned conductivity values are listed in Supplementary Table S1 in the Supplementary Material.

#### 2.3.4 Purkinje activation

**FIGURE 2:**
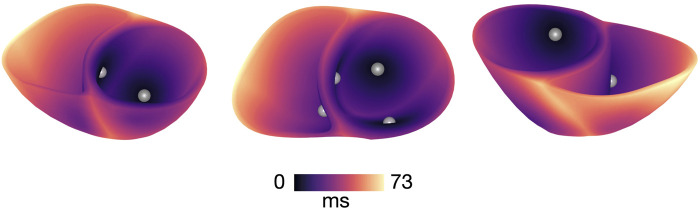
Activation map of sinus stimulation in a model with healthy conductivities and ${APD}_{\mathrm{206-290}}$ . Activation is initiated from four root points on the endo- and epicardium which simulate the Purkinje Network. The root points are indicated by the grey circles.

We simulated Purkinje activation by stimulating from four root points on the endocardium based on previous work (Cranford et al., 2018), as illustrated in Figure 2. Each point, defined as a spherical volume of 1 mm radius, was stimulated with a transmembrane current of 100 μ A of 1 ms duration. The three root points on the left ventricle and septum were activated simultaneously, and the right ventricular root point 5 ms later. To represent the fast-conducting Purkinje system, we assigned an isotropic CV of 2 m/s to the endocardial layer in all models (Gillette et al., 2023). The tuned conductivity values used for simulations are given in Supplementary Table S1 in the Supplementary Material. The resulting activation propagated from endocardium to epicardium, lastly activating the right ventricular base (Figure 2), consistent with experimental measurements (Durrer et al., 1970; Opthof et al., 2017). In our models with healthy tissue, the time from sinus onset to full ventricular activation was 72 ms, which is within the normal clinical range of 60–100 ms (Durrer et al., 1970; Konofagou and Provost, 2012; Opthof et al., 2017). For models with reduced conductivity (Section 2.3.3), the resulting times to full ventricular activation ranged between 74 and 105 ms in models with minimum and maximum CV slowing, respectively.

#### 2.3.5 SAC time course

In accordance with previous work (Hu et al., 2013), we used a simple representation of SAC where the current is given by:

$$I_{\text{SAC}}=g_{\text{SAC}}\left(V_{\text{m}}-E_{\text{SAC}}\right),$$ (3)

where $g_{\text{SAC}}$ is a time varying SAC conductance, $V_{\text{m}}$ is the transmembrane potential and $E_{\text{SAC}}$ is the SAC reversal potential. The term $I_{\text{SAC}}$ was added to the total sum of membrane ionic currents ( $I_{\text{ion}}$ in Equation (1)). We therefore created models with $E_{\text{SAC}}$ of −10, −20, −30, −40, −50, −60 and −70 mV to investigate how reentry depended on $E_{\text{SAC}}$ (Table 1). These values are within the range previously used in simulation studies (Trayanova et al., 2004; Hu et al., 2013; Hazim et al., 2021; Salvador et al., 2022).

The opening probability of SAC channels responds instantaneously to stretch (Reed et al., 2014) and increases with stretch amplitude (Pueyo et al., 2016; Gerach and Loewe, 2024). Thus, in the present study, we used a time dependent $g_{\text{SAC}}$ to represent the time course of stretch due to PM traction. The parameter $g_{\text{SAC}}$ was modeled by

$$\begin{array}{c} \frac{dg_{\text{SAC}}}{dt}=\frac{-\left(g_{\text{SAC}}-g_{\text{SACtarget}}\right)}{\tau_{\text{SAC}}}, \end{array}$$ (4)

where $g_{\text{SACtarget}}$ is a target conductance and $\tau_{\text{SAC}}$ is a time constant. The onset of SAC activation was varied in 1 ms steps, from 0 to 350 ms after sinus pacing (see Section 2.6.3). At time of SAC activation, $g_{\text{SACtarget}}$ was updated from 0 to 0.1 mS/ μ F, the latter representing maximum SAC conductance. The value 0.1 mS/ μ F, which is at the upper limit of those used in previous simulation studies with similar SAC representation (Trayanova et al., 2004), was chosen because we wanted to study effects of excessive traction on the myocardium. When $g_{\text{SAC}}$ reached a value close (within 10^−13^) to 0.1 mS/ μ F, $g_{\text{SACtarget}}$ was again updated to equal 0 mS/ μ F, resulting in a gradual deactivation of the SAC current. The total time profile of the SAC conductance is shown in Figure 1B. The same SAC activation (amplitude and time course) was used for the entire SAC region.

The value of $\tau_{\text{SAC}}$ , determining the length of SAC activation, was initially varied to yield pulse lengths of 10, 30, 50, 70 and 100 ms in an example model (model M1, Table 1). Since the duration of potential SAC activation caused by PM traction is unknown, we chose the value of $\tau_{\text{SAC}}$ which gave the longest vulnerability window for reentry (see Section 2.6.3). The vulnerability windows were 2 ms for pulse lengths 30, 50 and 70 ms, compared to 1 ms for pulse lengths of 10 and 100 ms. Thus, we chose a medium value of 50 ms, corresponding to $\tau_{\text{SAC}}$ = 1.8 for the remaining simulations.

### 2.4 Fibrotic microstructure

**FIGURE 3:**
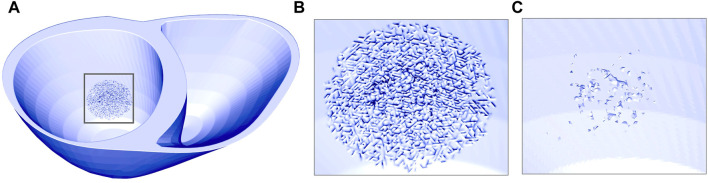
Ventricular mesh with fibrotic microstructure in the SAC region represented by mesh splitting. **(A)** and **(B)** illustrates the mesh surfaces. **(C)** Shows the elements which were completely isolated after mesh splitting and thus removed from the mesh.

In addition to representing fibrosis using conduction slowing, we created an additional set of models to investigate the effect of fibrotic microstructure. Fibrotic microstructure was applied within the SAC region, based on clinical reports of PM-related fibrosis in MVP and/or MAD (Dejgaard et al., 2018; Nagata et al., 2023). The microstructure was modeled using a mesh splitting approach previously implemented by Balaban et al. (2020). The algorithm creates electrically insulating clefts between mesh elements by duplicating individual mesh facets based on a given probability. We defined this probability as:

p=a|cosθ|, (5)

where θ is the angle between the element side and the fiber sheet normal direction. The faced-based parameter, a , was introduced to scale the probability such that the cleft density increased from the outer to the inner SAC region. To define a , we first mapped the element-based SAC region tags (Section 2.2) onto each facet *via* the mesh coordinates. The value of a was set to increase linearly from 0.1 in the outermost SAC layer to one in the innermost layer. The resulting geometrical model is shown in Figure 3. In total, we created a set of seven models with the same fibrotic microstructure, one for each $E_{\text{SAC}}$ between −10 and −70 mV, inclusive.

### 2.5 Single AP recordings

To investigate the effect of SAC activation on a single AP, we conducted simulations on a single element mesh in openCARP (open Cardiac Arrhythmia Research Package) (Plank et al., 2021; openCARP consortium et al., 2023). Using the SAC pulse defined in Section 2.3.5, we measured the effect of SAC activation at different time points relative to the AP of an example cell. The simulations were initiated from a prepaced state equal to that used in our ${APD}_{\mathrm{206-290}}$ ventricular models. Like in the full ventricular simulations, we tested $E_{\text{SAC}}$ in steps of 10 mV, from −10 to −70 mV. SAC was activated at four different time steps corresponding to phase 1-4 of the AP: at 6, 150, 270 and 330 ms after AP initiation.

### 2.6 Whole ventricular simulations

We conducted full ventricular model simulations in openCARP (Plank et al. (2021); openCARP consortium et al. (2023)) for all parameter combinations given in Table 1 and Supplementary Table S2 in Supplementary Material, equaling a total of 82 models. We first initialized our ventricular simulations from a single cell steady state, before further prepacing the entire model across three sinus beats. The single cell steady state was obtained by pacing a single cell 50 times, using the same basic cycle length (BCL) and ionic model as in the full ventricular simulation. Since the value of $I_{\text{Ks}}$ and thus APDs differed throughout the ventricle (see Section 2.3.2), we selected an example cell for this initialization: for ventricular models with ${APD}_{\mathrm{241-290}}$ and ${APD}_{\mathrm{206-290}}$ , we used the default value of $I_{\text{Ks}}$ (APD = 290 ms). For models with ${APD}_{\mathrm{188-235}}$ , in which $I_{\text{Ks}}$ was scaled for all cells, we used a scaling factor of 3.5 (APD = 214 ms). As the cell model states used to initialize the full ventricular simulation did not account for differences in $I_{\text{Ks}}$ , we further prepaced our entire model for three consecutive sinus beats with the chosen BCL, similar to prepacing cycles used in previous work (Balaban et al., 2020). The delay between the last sinus beat and SAC activation was then systematically varied in order to identify vulnerability windows for arrhythmia (see Section 2.6.3).

We used a BCL of 500 ms as a baseline for our simulations (Table 1). However, to investigate how the simulation results could vary depending on the selected heart rate, we additionally included a set of simulations for BCL of 1,000 ms [see Supplementary Material (Supplementary Table S2)].

#### 2.6.1 Extracellular potential recordings

Throughout our simulations, we recorded the extracellular potential in a point corresponding to the left shoulder. This point was determined by first orienting our ventricular mesh according to a previously generated cardiac mesh with surrounding torso (Uv et al., 2022). A point on the left shoulder (see Supplementary Figure S1) of the torso was then selected manually using ParaView (Ayachit, 2015). The extracellular potential was calculated using the $\Phi_{\text{e}}$ recovery method for monodomain simulations as previously used (Bishop and Plank, 2011) and implemented in openCARP.

#### 2.6.2 Definition of reentry

We aimed to investigate conditions which could result in a globalized arrhythmia, rather than a small local reactivation of tissue. To achieve this we recorded a reentry in our simulations as a global reactivation of the myocardium following external stimuli (sinus pacing) or ectopic activity (SAC-induced depolarization). Global reactivation was visually determined as a spike in extracellular potential (Figures 6, 7). In some cases, the activation wave could propagate back into an early repolarized SAC region without subsequent escape into the surrounding myocardium, and consequently, with no visible spike in the recorded extracellular potential. By our definition, these cases were not considered as cases of reentry, as global reactivation did not occur. We classified reentry as sustained if there was still reentrant activity 1,000 ms after the last sinus activation and unsustained otherwise.

#### 2.6.3 Determination of vulnerability windows for reentry and ectopy

To identify potential vulnerability windows for reentry, we varied the delay between the last sinus pacing and the activation of the SAC region in 1 ms intervals. When exploring how the reentrant mechanism and vulnerability window varied as a function of $E_{\text{SAC}}$ in healthy models and in models with fibrotic microstructure (M1-M7 and M15-M21, Table 1), we varied the delay between 1 and 350 ms in 1 ms intervals, covering the time period from sinus activation to the end of the T-wave.

When investigating the effect of varying size of the SAC region, CV and APD heterogeneity, we used the vulnerability windows identified previously (M1-M7, Table 1) as starting points when searching for reentry. For this purpose, we again varied the SAC onset in steps of 1 ms until either 1) both the beginning and end of a vulnerability window was identified or 2) all time points within the period of 1–350 ms after sinus activation had been investigated. In case 1), we additionally searched within 5 ms on each side of the vulnerability window to check that reentry was not induced within these periods. Furthermore, in models where SAC triggered ectopy through local depolarization ( $E_{\text{SAC}}$ from −40 to −70 mV), we also varied the SAC activation in steps of 1 ms until the end of the T-wave (350 ms) to identify at which time SAC onset triggered ectopy.

## 3 Results

### 3.1 Effect of SAC reversal potential in single cells

**FIGURE 4:**
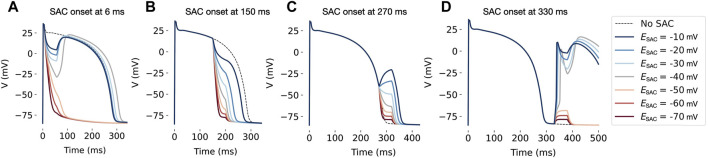
Effect of SAC activation in a single cell, with duration of 50 ms and onset at various time points during the AP. In all models, SAC has a repolarizing effect when applied at 6 ms **(A)** and 150 ms **(B)** after AP initiation. When onset is at 270 ms **(C)**, models with $E_{\text{SAC}}$ = −10 and −20 mV experience a depolarizing effect and all models experience an increased APD. At 330 ms **(D)**, SAC onset triggers another AP in models with $E_{\text{SAC}}$ between −10 and −40 mV. Dotted line shows a reference AP without SAC. Values of $E_{\text{SAC}}$ are in mV. AP: action potential. SAC: Stretch activated channels.

The effect of varying $E_{\text{SAC}}$ and the timing of SAC onset on a single AP is demonstrated in Figure 4. When onset during the initial repolarization (phase 1, 6 ms after AP initiation), SAC activation had a repolarizing effect for all $E_{\text{SAC}}$ (Figure 4A). For $E_{\text{SAC}}$ between −10 and −40 mV, the effect was transient and followed by a depolarization when SAC was switched off. For $E_{\text{SAC}}$ between −50 and −70 mV, SAC activation caused a rapid, complete repolarization and thus extensive shortening of the APD. During the AP plateau (phase 2, 150 ms after AP initiation), the repolarizing effect of SAC lead to APD shortening in all models (Figure 4B). If SAC is onset 120 ms later, during the last phase of repolarization (phase 3, 270 ms after AP initiation), a depolarizing effect is observed for $E_{\text{SAC}}$ of −10 and −20 mV (Figure 4C). Finally, when onset after reaching resting potential (phase 4, 330 ms after AP initiation) SAC has a depolarizing effect in all models (Figure 4D). In the latter case, a secondary AP is triggered for $E_{\text{SAC}}$ from −10 and −40 mV.

### 3.2 Effect of SAC reversal potential on reentry in ventricular models

**FIGURE 5:**
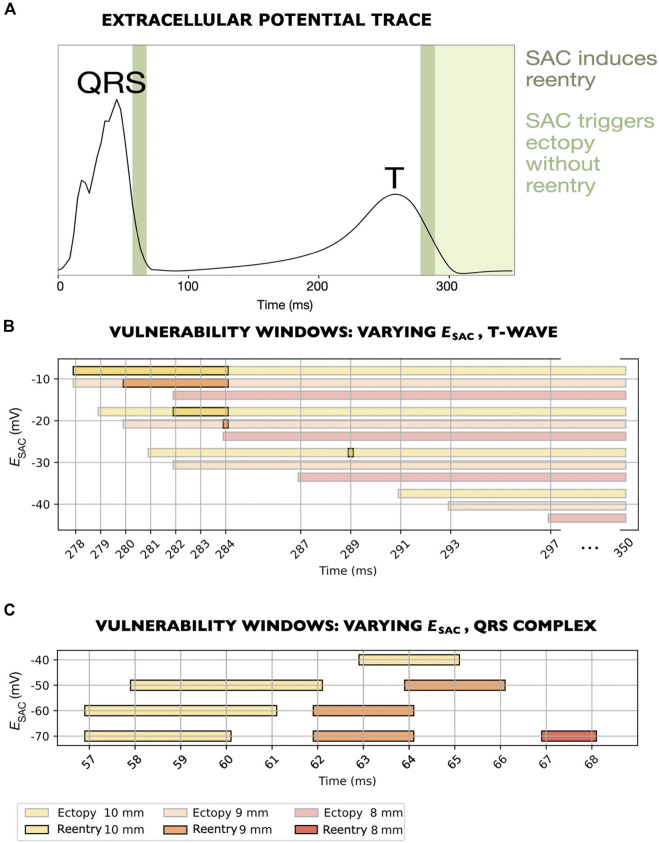
Vulnerability windows for reentry across various levels of $E_{\text{SAC}}$ (M1-M7 models, Table1). **(A)** Extracellular potential trace recorded from a single electrode at the left shoulder. Time is measured from the last sinus pacing. Dark green panels mark the time periods within which SAC onset triggered reentry. Light green panel indicates instances where SAC triggered ectopy without reentry (last point measured at 350 ms). Notches in the QRS complex arise from the activation sequence where the endocardium is activated *via* four root points based on Cranford et al. (2018), rather than stimulating the entire endocardium simultaneously. **(B)** For $E_{\text{SAC}}$ between −10 and −30 mV, all vulnerability windows overlap with the T-wave. The time period within which reentry occurred (278–289 ms) correspond to the right dark green panel in **(A)**. **(C)** For $E_{\text{SAC}}$ between −40 and −70 mV, all vulnerability windows overlap with the QRS complex. The plotted time period (57–68 ms) correspond to the left dark green panel in **(A)**. All reentries for the models considered (M1-M7) were unsustained.

We explored the effect of varying $E_{\text{SAC}}$ in our 3D ventricular models when activating SAC at the ventricular PM insertion point (models M1-M7, Table 1). In the resulting simulations, we observed two separate vulnerable periods for arrhythmia: close to the end of the QRS complex and during the down stroke of the T-wave (Figure 5A). Depending on the value of $E_{\text{SAC}}$ , SAC activation during one of these periods could induce reentry. In these models (M1-M7), all reentries were unsustained. Each of the two vulnerable periods were associated with a distinct mechanism leading to reentry (Section 3.4.1, Section 3.4.2). For each considered value of $E_{\text{SAC}}$ , we observed only one of these mechanisms.

We observed one of the reentrant mechanism in models with $E_{\text{SAC}}$ of −10, −20 and −30 mV (M1-M3 models, Table 1). The vulnerability windows all aligned with the down stroke of the T-wave (Figures 5A, B). In this set of models, reentry was initiated due to local depolarizations triggered by SAC activation (Section 3.4.1).

The second mechanism was observed in the remaining models, with $E_{\text{SAC}}$ of −40, −50, −60 and −70 mV (M4-M7 models, Table 1). The vulnerability windows were near the end of the QRS complex (Figures 5A, C). The reentries were formed due to early repolarization in the SAC region (Section 3.4.2).

In one of the models where reentry was triggered by early repolarization ( $E_{\text{SAC}}$ = −40 mV, M4 model, Table 1), SAC could also have a depolarizing effect which was strong enough to trigger ectopy (see Supplementary Video S8). The latter was observed for SAC onset close to the end of the T-wave or later (from 291 ms after sinus activation and onwards, Figure 5B, bottom row). However, these ectopic beats did not lead to reentry. Similarly, in models with $E_{\text{SAC}}$ of −10, −20 and −30 mV, SAC triggered an ectopic beat without reentry if SAC was activated either after the end of the vulnerability window, or up to 8 ms before the start of the vulnerability window (Figures 5A, B). In models with $E_{\text{SAC}}$ of −70, −60 and −50 mV, on the other hand, the depolarizing effect of SAC was not sufficient to trigger ectopy at any point during the cardiac cycle.

### 3.3 Effect of size of SAC region

For all values of $E_{\text{SAC}}$ , we tested the effect of varying the size of the SAC region. With a SAC radius of 10 mm, reentry could be induced in all models (Figure 5). When decreasing the radius to 9 mm, the vulnerability windows were either narrowed (for $E_{\text{SAC}}$ of −10, −20, −50, −60 and −70 mV, Figures 5B, C) or reentry could no longer be induced (for $E_{\text{SAC}}$ of −30 and −40 mV, Figure 5B).

Decreasing the SAC radius from 10 to 9 mm also slightly affected which times of SAC onset that triggered ectopy, although the effect was small: The earliest SAC onsets which triggered ectopy were shifted from 279 to 280 ms, from 281 to 282 ms and from 291 to 293 ms in models with $E_{\text{SAC}}$ of −20, −30 and −40 mV, respectively (Figure 5B). For $E_{\text{SAC}}$ of −10 mV, the corresponding time was 278 ms in both models.

A further decrease in SAC radius from 9 to 8 mm rendered six of the seven models non-inducible to reentry. Only for $E_{\text{SAC}}$ of −70 mV did we still observe a vulnerability window, at 67–68 ms after sinus pacing (Figure 5C, bottom row). Furthermore, when decreasing the radius from 9 to 8 mm, the earliest SAC onsets which triggered an ectopic beat were further shifted to slightly later time points in the cardiac cycle: From 278 to 282 ms, from 280 to 284 ms, from 282 to 287 ms and from 293 to 297 ms after sinus pacing for $E_{\text{SAC}}$ of −10, −20, −30 and −40 mV, respectively (Figure 5B). In the model with reentry for 8 mm SAC radius ( $E_{\text{SAC}}$ of −70 mV), a final decrease from 8 to 7 mm made the model non-inducible to reentry.

### 3.4 Reentrant mechanisms

#### 3.4.1 SAC activation during the T-wave

**FIGURE 6:**
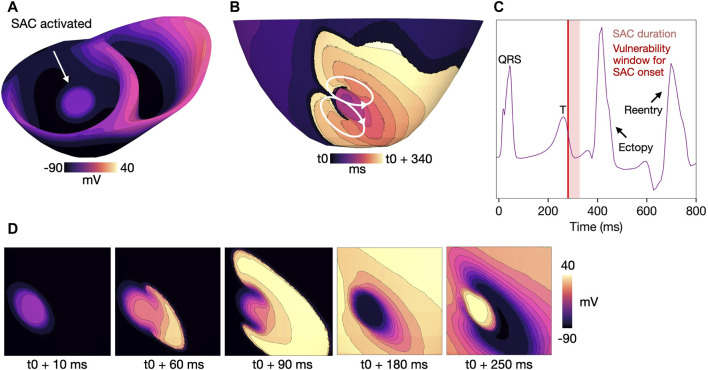
Example reentry in a model with $E_{\text{SAC}}$ = −10mV, 500 ms BCL, healthy conductivities and a SAC region of 10 mm radius (model M1, Table 1). SAC onset is at 278 ms after start of sinus activation. **(A)** Voltage map at 10 ms after SAC onset, showing a depolarized SAC region. **(B)** Activation map showing the reentrant circuit which forms when the SAC-induced activation escapes the SAC region, with t0 indicating the time of SAC onset. **(C)** Extracellular potential recorded from a point on the left shoulder during the same simulation. The light red panel mark the total duration of SAC activation when onset is at 278 ms. The vulnerability window for which simulations resulted in reentry (SAC onset 278–284 ms) is overlaid (thin, dark red panel). The T-wave is followed by two spikes in extracellular potential: the first is an ectopic beat triggered by SAC-induced depolarization and the second is caused by the reentrant wave (see arrows). Time is measured from start of sinus activation. **(D)** Zoomed in voltage maps of the same reentry and the same viewing angle as in **(B)** at time points 10, 60, 100, 180 and 250 ms after SAC activation (t0). A movie of the reentry is given in Supplementary Video S7. SAC: Stretch activated channels. BCL: basic cycle length.

The mechanism of reentry caused by local, SAC-induced depolarization in models M1-M3 (Table 1) is illustrated in Figure 6 (M1, Table 1). In sufficiently repolarized tissue and high enough $E_{\text{SAC}}$ ( >= −40 mV), SAC activation increases the membrane potential and triggers ectopic activity. In our illustrative example (M1, Table 1), SAC is activated from 278 ms after onset of the last sinus pacing (Figures 6A, D). At this time point, the myocardium is partly repolarized, coinciding with the down stroke of the T-wave (Figure 6C). The activation then escapes from the SAC region towards the side of the myocardium which is first to repolarize. Subsequently, the wave spreads towards the side which is the last to repolarize. In models with $E_{\text{SAC}}$ of −10 to −30 mV, this type of propagation (activation spreading anisotropically from the SAC region) resulted in a reentrant circuit, with a figure-of-eight pattern which was visible when viewed from either the endocardial or epicardial side (Figure 6B). Example videos of the reentry initiation and propagation for each value of $E_{\text{SAC}}$ demonstrating the above-mentioned mechanism are given in the Supplementary Material (Supplementary Video S5–S7).

#### 3.4.2 SAC activation during QRS complex

**FIGURE 7:**
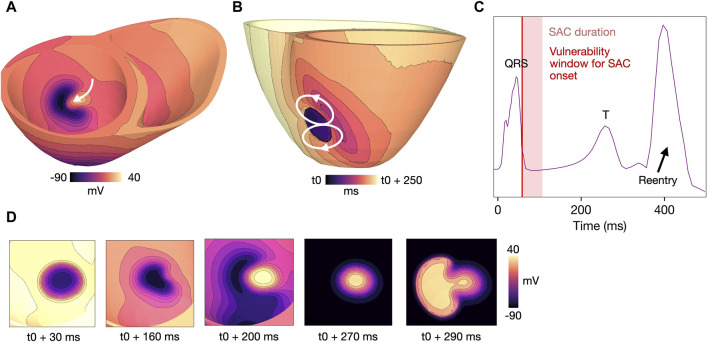
Example reentry for a model with $E_{\text{SAC}}$ = −70 mV, ${APD}_{\mathrm{206-290}}$ , 500 ms BCL, healthy conductivities and a SAC region of 10 mm radius (model M7, Table 1). SAC onset is at 57 ms after start of sinus activation. **(A)** Voltage map of the sinus activation wave propagating back into the early repolarized SAC region at 170 ms after SAC onset. **(B)** Activation map showing the reentrant circuit which forms after the propagating wave subsequently escapes the SAC region, with t0 indicating the time of SAC onset. **(C)** Extracellular potential recorded during the same simulation with the period of SAC activation (light red panel). The vulnerability window for which simulations resulted in reentry (SAC onset 57–60 ms) is overlaid (thin, dark red panel). The ventricular contraction caused by the reentrant wave is indicated by the arrow. Time is measured from start of sinus activation. **(D)** Zoomed in voltage maps showing formation of the reentry, viewed from the same angle as in **(A)**. The snapshots show early repolarization in the SAC region (at 30 and 60 ms), propagation of the sinus wave back into the SAC region (200 and 270 ms) and finally, the activation wave reentering into surrounding myocardium (290 ms). A movie of the reentry is given in Supplementary Video S1. SAC: Stretch activated channels. BCL: basic cycle length.

Reentry was initiated due to early repolarization in the SAC region in models M4-M7 ( $E_{\text{SAC}}$ from −40 to −70 mV, Table 1). Figure 7 shows the mechanism of reentry initiation in an example model (M7, Table 1). The activation of SAC lowers the membrane potential locally, causing early repolarization of the SAC region compared to the surrounding myocardium. As a result, the surrounding myocardial tissue, still depolarized from the sinus activation, propagates back into the SAC region around 170 ms after SAC onset (Figure 7A). The activation within the SAC region subsequently escapes into the part of the surrounding tissue which is the first to repolarize, forming a reentrant circuit (Figure 7B). Snapshots showing the reentry formation are presented in Figure 7D. The peak in extracellular potential (Figure 7C) corresponds to the time when the entire myocardium is reactivated by the reentrant wave. For models with $E_{\text{SAC}}$ from −40 to −70 mV (M4-M7, Table 1), all reentries followed the same type of mechanism: early repolarization of tissue in the SAC region caused the activation wave to propagate back into this region, before escaping into surrounding tissue. As in Section 3.4.1, a figure-of-eight circuit could be observed on either the endocardial or epicardial surface. Example videos of reentries for all $E_{\text{SAC}}$ from −40 to −70 mV are given in Supplementary Material (Supplementary Videos S1–S4).

### 3.5 Effect of conduction slowing

We investigated how conduction slowing affected reentry due to both SAC-induced depolarization and early repolarization. We performed simulations for all $E_{\text{SAC}}$ , using a SAC radius of 10 mm and ${APD}_{\mathrm{206-290}}$ heterogeneity (see Methods and Materials Section 2.3.2).

#### 3.5.1 Global CV slowing

**FIGURE 8:**
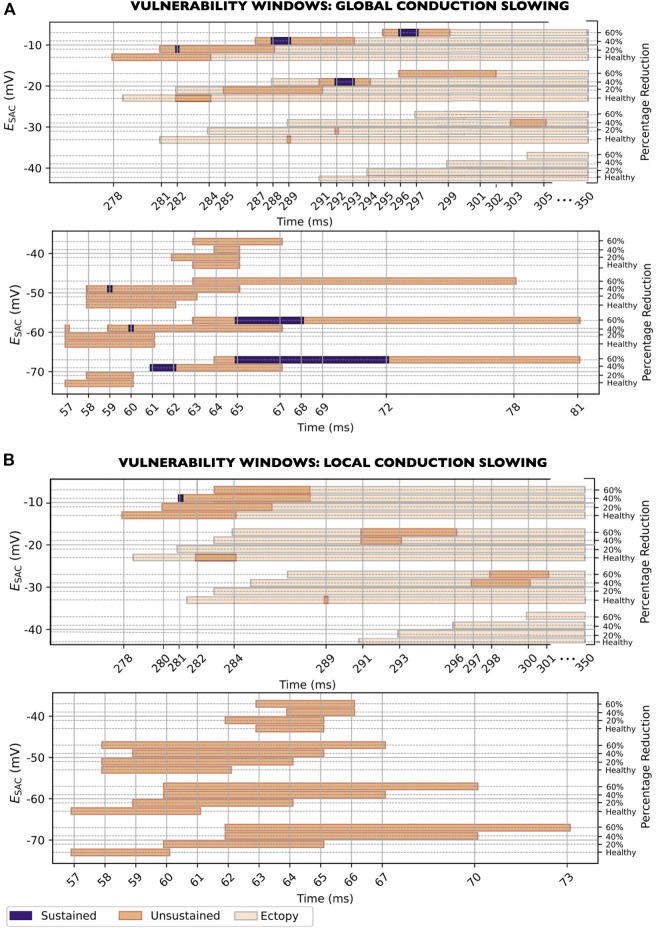
Vulnerability windows for reentry across all levels of $E_{\text{SAC}}$ , for global **(A)** and local **(B)** CV slowing (models M8-M14, Table 1). Three degrees of slowing were investigated: 20, 40% and 60% CV reduction. CV: Conduction velocity.

When reducing the CV globally (models ${M8-M14}_{20/40/60,\text{g}}$ , Table 1), sustained reentry could be induced in 9/21 (43%) of models (Figure 8). The vulnerability windows for reentry for all models are shown in Figure 8. The beginning of the vulnerability windows were generally shifted towards later time points in the cardiac cycle as CV slowing was increased. However, the effects were small, with the largest shift being 17 ms: from 278 ms to 295 ms after previous sinus pacing in models with $E_{\text{SAC}}$ of −10 mV (models M1 vs. ${M8}_{60,g}$ in Table 1). In comparison, for $E_{\text{SAC}}$ of −40 mV, there was no difference between the start of the vulnerability window for healthy CV (M4) and maximum CV slowing ( ${M11}_{60,g}$ ).

Regarding the width of the vulnerability windows, global CV slowing increased the duration of the vulnerability windows for most $E_{\text{SAC}}$ : When compared to models with healthy CV, maximum CV slowing increased the length of the vulnerability windows from 3 to 7 ms, from 3 to 5 ms, from 5 to 16 ms, from 5 to 19 ms and from 4 to 18 ms for $E_{\text{SAC}}$ of −20, −40, −50, −60 and −70 mV, respectively. However, in one case, the vulnerability windows were slightly shorter in models with maximum CV slowing compared to in healthy models (5 vs. 7 ms for $E_{\text{SAC}}$ of −10 mV). Furthermore, for $E_{\text{SAC}}$ of −30 mV, maximum CV slowing rendered the model non-inducible to reentry. Notably, in one of the models ( $E_{\text{SAC}}$ of −60 mV and 40% reduced CV), we observed a 1 ms gap in the vulnerability window, at 58 ms after previous sinus pacing, where reentry was not induced.

#### 3.5.2 CV slowing in the SAC region

When reducing the CV in the SAC region only (models ${M8-M14}_{20/40/60,\text{l}}$ , Table 1), sustained reentry was induced in 1/21 (5%) of models (Figure 8). The vulnerability windows for reentry across all $E_{\text{SAC}}$ are shown in Figure 8. The beginning of the vulnerability windows were in most cases shifted by a small amount, to later time points in the cardiac cycle: from healthy CV to maximum CV slowing, the beginning of each window was shifted from 278 to 283 ms (5 ms), from 282 to 291 ms (9 ms), from 289 to 298 ms (9 ms), from 57 to 60 ms (3 ms) and from 57 to 62 ms (5 ms) after the previous sinus pacing in models with $E_{\text{SAC}}$ of −10, −20, −30, −60 and −70 mV, respectively. For $E_{\text{SAC}}$ of −40 and −50 mV, the beginning of the vulnerability windows were the same for healthy CV and maximum CV slowing.

The width of the vulnerability windows differed only slightly when changing from healthy CV to maximum CV slowing: from 7 to 6 ms, from 3 to 6 ms, from 1 to 4 ms, from 3 to 4 ms, from 5 to 10 ms, from 5 to 11 ms and from 4 to 12 ms for $E_{\text{SAC}}$ of −10, −20, −30, −40, −50, −60 and −70 mV, respectively. In two of the models with reduced CV in the SAC region ( $E_{\text{SAC}}$ of −20 and −30 mV, both with 20% CV reduction), reentry could not be induced for SAC onset at any time point in the cardiac cycle.

### 3.6 Effect of fibrotic microstructure

**FIGURE 9:**
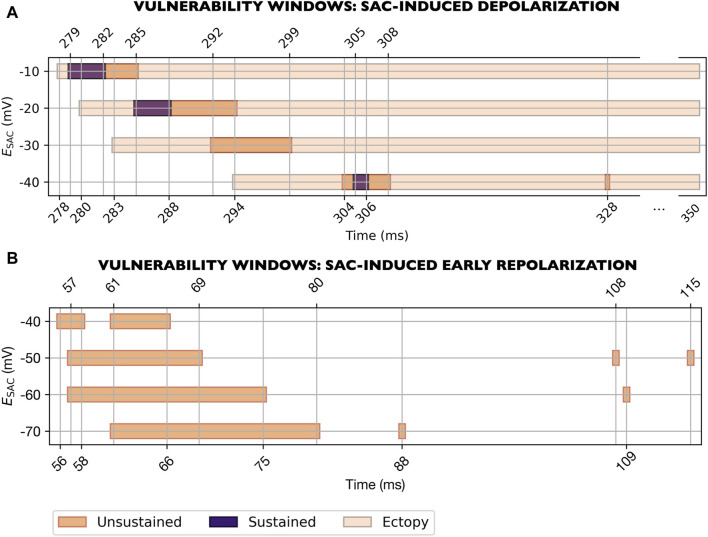
Vulnerability windows for reentry for models with fibrotic microstructure in the SAC region (M15-M21, Table 1). Results for all levels of $E_{\text{SAC}}$ are shown. Reentry was triggered by SAC-induced depolarization **(A)** or SAC-induced early repolarization **(B)**.

**FIGURE 10:**
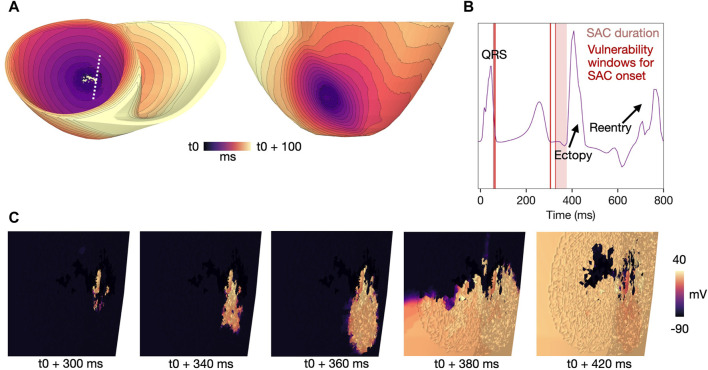
Example of a micro-reentry induced in a model with fibrotic microstructure for $E_{\text{SAC}}$ of −40 mV and SAC onset at 328 ms. **(A)** voltage map of the ectopy. The time of SAC onset is represented as t0. The white stapled line marks the cross-section of myocardium presented in **(C)**. **(B)** Extracellular potential trace of the last sinus pacing and following reentry when SAC was activated from 328 ms. Recordings were made from a single electrode corresponding to the left shoulder. The light red panel marks the duration of SAC activation for the recorded simulation. The dark red lines marks all vulnerability windows for the model. Arrows mark the ectopic beat triggered by SAC activation and the following reentrant wave. Time is measured from the start of sinus activation. **(C)** Voltage maps showing a cross-section of the model, as indicated by the white stapled line in **(A)**. t0 indicates the time of SAC activation. SAC: Stretch activated channels. BCL: basic cycle length.

To investigate how fibrotic microstructure affected reentry vulnerability, we represented fibrosis in the SAC region using mesh splitting (see Methods and Materials Section 2.4). In these models, 10/96 (10%) reentries were sustained (Figure 9). Figure 9A presents the vulnerability windows for reentry triggered by SAC-induced depolarization. These reentries were observed for $E_{\text{SAC}}$ of −10 to −40 mV. As for models without fibrosis, the vulnerability windows for reentry caused by SAC-induced depolarization occurred primarily during the down slope of the T-wave interval (279–308 ms after sinus pacing). Activation maps for an example reentry induced by SAC onset at 279 ms is given in Supplementary Figure S4 in the Supplementary Material. In contrast to models without fibrotic microstructure, however, we also observed a single reentry at 328 ms after sinus pacing (Figure 9A, $E_{\text{SAC}}$ of −40 mV). This reentry, a micro-reentry with a focal-like activation pattern, was formed when activation was trapped within the fibrotic microstructure. This allowed the surrounding tissue to fully repolarize, leading to focal-like propagation when the activity escaped from the dense cleft region. Activation and voltage maps of the reentry, along with an extracellular potential trace of the simulation, is given in Figure 10.

**FIGURE 11:**
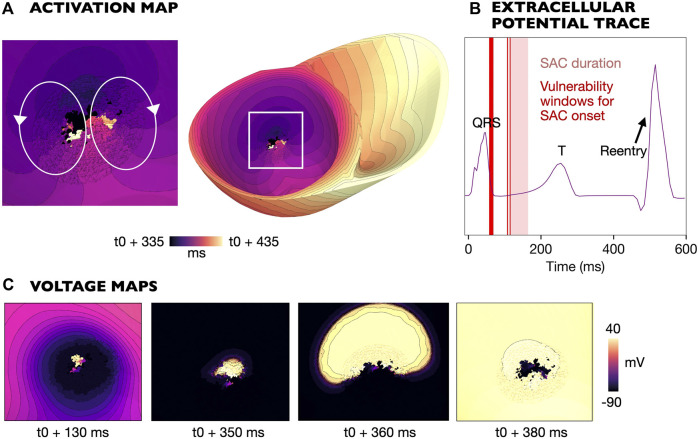
Example of a micro-reentry induced in a model with fibrotic microstructure and $E_{\text{SAC}}$ of −50 mV for SAC onset at 115 ms. **(A)** Voltage map of the reentry. The time of SAC onset is represented as t0. **(B)** Extracellular potential trace of the last sinus pacing and following reentry when SAC was activated from 115 ms. Recordings were made from a single electrode corresponding to the left shoulder. The light red panel marks the duration of SAC activation for the recorded simulation. The dark red lines marks all vulnerability windows for the model. Time is measured from the start of sinus activation. **(C)** Voltage maps showing formation of the reentry. t0 indicates the time of SAC activation. SAC: Stretch activated channels. BCL: basic cycle length.

Figure 9B shows the vulnerability windows for reentry triggered by SAC-induced early repolarization. These reentries were observed in models with $E_{\text{SAC}}$ of −40 to −70 mV. The vulnerability windows were clustered around 56–80 ms, close to the down slope of the QRS complex, as in models without fibrotic microstructure. However, when fibrotic microstructure was incorporated in the models, we observed single cases of reentry at 88, 108, 109 and 115 ms after the last sinus pacing. These were micro-reentries caused by the heterogeneous activation and repolarization of the SAC region. Figure 11 shows one of these micro-reentries, triggered by SAC onset at 115 ms after pacing. In total, 13/96 reentries (14%) in models with microstructure were triggered outside the most vulnerable periods (namely, the down slope of the T-wave (see previous results in section 3.4.1; Figures 5A, B) or down slope of the QRS-complex [see previous results in section 3.4.2; Figures 5A, C)]. However, only four of the reentries (4%) were more than 10 ms outside of these vulnerable periods on extracellular potential recordings.

## 4 Discussion

In the present study, we demonstrated that stretch through SAC activation of the PM insertion region could induce reentry in an image-based model of a patient with MVP and MAD. Two main mechanisms of reentry were identified: 1) SAC-induced depolarization and 2) SAC-induced early repolarization. For both mechanisms of reentry initiation, the activation of SAC could lead to a tissue state wherein the SAC-region was activated whilst the surrounding myocardium was partly repolarized, as illustrated in Figure 6A. From this state, the wavefront within the SAC region could escape and form a reentrant figure-of-eight circuit (Figures 6B, 7B). Additionally, in models with fibrotic microstructure, SAC-induced depolarization and SAC-induced early repolarization could both trigger the formation of micro-reentry originating from the SAC region (Figures 10, 11). To summarize, the key findings of this study are:

In models with $E_{\text{SAC}}$ −10 to −30 mV without fibrotic microstructure, SAC activation in the PM insertion region could trigger depolarizations and reentry if activated during the down stroke of the T-wave (Figures 5A, B). In models with $E_{\text{SAC}}$ −40 to −70 mV and without fibrotic microstructure, SAC activation in the same region could cause early repolarization and subsequent reentry when applied during the down stroke of the QRS complex (Figures 5A, C). Fibrotic microstructure in the SAC region could give rise to micro-reentry. 14% of reentries in these models were triggered by SAC activation outside of the above-mentioned periods (Figure 9). Reentry required a relatively large region of stretch surrounding the PM insertion point ( >= 8 mm radius in models with healthy tissue, Figure 5). Reentry could be induced in models both with and without fibrosis (Figure 8).

### 4.1 Timing of stretch and reentry vulnerability window

Timing of the stretch by SAC activation was a key component to whether reentry was induced. The time with respect to the previous sinus activation determines the degree of repolarization within and around the SAC region, and therefore whether a reentrant circuit is able to form. In the present study, the vulnerability windows were short (1–20 ms) and generally coinciding with either the end of the QRS complex ( $E_{\text{SAC}}$ of −70 to −40 mV) or end of the T-wave ( $E_{\text{SAC}}$ of −30 to −10 mV in models without fibrotic microstructure and $E_{\text{SAC}}$ of −40 to −10 mV in models with fibrotic microstructure). Similarly, others have identified vulnerability windows of 10–20 ms for reentry due to stretch-induced ectopy in the setting of commotio cordis in rabbits (Li et al., 2004; Quinn et al., 2017). In these studies, arrhythmia was induced if the impact overlapped with tissue which was partly repolarized. Consistent with these findings, we observed that when the SAC region is depolarized, a reentrant circuit could form in the absence of anatomical barriers if the surrounding tissue is partly repolarized, since the non-repolarized tissue acts as a functional conduction block.

In swine, ventricular fibrillation could be induced by mechanical impact delivered in the period of 40–1 ms before the peak of the T-wave [Link (2003; 2012)]. During this period, the authors reported a clear increase in impact-induced arrhythmia, even though impacts during the QRS or ST segment could occasionally cause nonsustained ventricular tachycardia. While the above studies point to the T-wave as the most vulnerable period for stretch-induced reentry, we identified vulnerability windows both during the T-wave and the QRS complex. In terms of patients with MVP, further investigation is required to determine the most vulnerable period for arrhythmia induction. Specifically, vulnerability for reentry would depend on potentially patient-specific SAC dynamics and periods of abnormal stretch throughout the cardiac cycle. Patient electrophysiological measurements or strain-profiles (Huttin et al., 2017) may be incorporated into ventricular models in order to move computational investigations on MVP patients from the general to the patient-specific mechanisms of reentry.

Since SAC activate immediately after stretch of the affected tissue (Reed et al., 2014; Quinn and Kohl, 2021), clinically-measured timings of stretch can offer insight as to when SAC are likely to be activated in MVP patients. With respect to the cardiac cycle, abnormal stretch of the PMs and adjacent myocardium have been clinically observed at different time points. Studies have measured a superior shift of the PM tip (Sanfilippo et al., 1992) and an abnormal increase in PM velocity (Han et al., 2010) in the period of mid-to end systole in patients with MVP. In patients presenting with late systolic MVP, reduced PM contraction from early to late systole has been measured (Hei et al., 2019). The findings of Nagata et al. (2023) suggested stretch of the basal myocardium around late-to end systole, an observation hypothesized to be caused by leaflet prolapse and excessive PM traction. Lastly, Huttin et al. (2017) showed that reduced myocardial strain, possibly related to abnormal PM traction, was present in early systole and late-to post systole. Late systole, consistently reported as a period of stretch or reduced contraction of the PM or adjacent myocardium in the above studies, corresponds with our vulnerability window during the T-wave. Sufficient stretch of the PM insertion region during late systole may give rise to the reentrant mechanism we observed (reentry caused by SAC-induced depolarization). Conversely, abnormal motion during early systole as described in the above studies could potentially trigger reentry through early repolarization, corresponding to the vulnerability window during the QRS complex observed in the present study. In our simulations, for both mechanisms of reentry initiation, the first ectopic beat occurred after the T-wave (Figures 6, 7), consistent with ECG recordings of PVCs originating from the PMs in MVP patients (Fulton et al., 2018).

### 4.2 SAC reversal potential determines reentrant mechanism

SAC are defined as a category of ion channels that open in response to stretch, usually divided into non-selective cation channels ( ${SAC}_{\text{ns}}$ ) and potassium selective channels ( ${SAC}_{\text{K}}$ ) (Kohl et al., 1999). A wide range of reversal potentials have been measured for the two classes (Hu and Sachs, 1997). Typical reported values are in the range −20 to 0 mV for ${SAC}_{\text{ns}}$ (Quinn and Kohl, 2021) and near the K^+^ reversal potential (around −90 mV) for ${SAC}_{\text{K}}$ (Kohl et al., 1999; Quinn and Kohl, 2011). The effective SAC reversal potential, here modeled as $E_{\text{SAC}}$ , is influenced by the relative amount of the different SAC channels active. Some have argued that compared to ${SAC}_{\text{K}}$ , ${SAC}_{\text{ns}}$ dominate the myocardial stretch response in healthy tissue (Quinn and Kohl, 2021). These suggested SAC dynamics may, however, change in cardiac disease (Reed et al., 2014; Quinn and Kohl, 2021). As the experimental evidence regarding the ratio of these different channel classes are inconclusive, we varied $E_{\text{SAC}}$ from −10 to −70 mV, which is within the range previously used in simulation studies (Trayanova et al., 2004; Hu et al., 2013; Hazim et al., 2021; Salvador et al., 2022). Consistent with our single-cell analysis, previous work have reported both repolarizing and depolarizing effects of SAC, depending on SAC reversal potential and the timing of SAC onset (Li et al., 2004).

Ectopy triggered by late systolic or diastolic stretch, known to occur experimentally (Gornick et al., 1986; Zabel et al., 1996; Quinn et al., 2017), was only observed in our models with $E_{\text{SAC}}$ between −40 and −10 mV. Depending on model choice and the exact timing of T-wave stretch, these ectopic beats could evolve into reentrant circuits. This range of $E_{\text{SAC}}$ represents a relatively large influence of ${SAC}_{\text{ns}}$ as compared to ${SAC}_{\text{K}}$ . Consistent with our findings, previous work has shown that mechanical stimuli delivered during the T-wave can induce ventricular fibrillation *via* activation of ${SAC}_{\text{ns}}$ in isolated rabbit hearts (Quinn et al., 2017). The proposed mechanism of reentry, as previously simulated (Garny and Kohl, 2004; Li et al., 2004), is in accordance with our observations: stretch of partly repolarized tissue leads to a reentrant circuit through the region of stretch, analogous to the reentry in Figure 6.

In the present study, only models with $E_{\text{SAC}}$ less than or equal to −40 mV, representing a considerable contribution from ${SAC}_{\text{K}}$ , gave rise to reentry due to early repolarization. The vulnerability window for these reentries were near or coinciding with the QRS complex, whereas experimental evidence has typically pointed to the T-wave as the most vulnerable period for arrhythmia (Li et al., 2004; Quinn and Kohl, 2011; Link, 2012).

In summary, stretch-induced depolarization mechanisms are more concordant with experimental reports in terms of induced ectopic activity and observed vulnerability windows. However, clinical validation of the two mechanisms (both SAC-induced depolarization and SAC-induced early repolarization) as potential triggers of reentry in MVP patients is needed.

### 4.3 APD heterogeneity and reentry

Previous simulation studies have shown that in a region with partly repolarized tissue, ectopic activity may form a reentrant wave around the temporary conduction block (Garny and Kohl, 2004; Li et al., 2004). Similarly, we found that after SAC activation, the wavefront moved out of the SAC region *via* the side of the myocardium which repolarized first towards the side repolarizing later, forming a reentrant circuit (Figures 6, 7).

Spatial differences in repolarization times across the myocardium are determined by the local activation times and APDs. Although reports differ in the direction and strength of the measured gradients, the presence of spatial heterogeneity in repolarization has been well documented (Janse et al., 2005; Glukhov et al., 2010; Boukens et al., 2015; Srinivasan et al., 2016). Both individual differences among patients as well as local gradients in various regions of the heart have been observed (Opthof et al., 2017). Repolarization times may also be altered in patients with cardiac disease, including patients with MVP (Digeos-Hasnier et al., 2005; Chauhan et al., 2006). Due to the large variation in reported repolarization gradients, we tested two additional APD gradients (see Materials and Methods) in two example models ( $E_{\text{SAC}}$ of −10 mV and $E_{\text{SAC}}$ of −70 mV), by varying the APD heterogeneity according to the above-mentioned literature. For these simulations, the type of reentrant mechanism observed (either SAC-induced early repolarization during the QRS complex or SAC-induced depolarization during the T-wave) was not affected by the changes in APD gradient. However, the choice of gradient did influence the duration of the vulnerability window as well as reentry inducibility and sustainability (See Supplementary Figure S2 in Supplementary Results). Similarly, previous experimental and computational work has showed that arrhythmic vulnerability depends on APD- and repolarization dispersion (Kuo et al., 1985; PAK et al., 2004; Chauhan et al., 2006; Avula et al., 2019).

### 4.4 Effect of size of the SAC region

Imaging and histological studies report a large variation in how the PM base is attached to the ventricular wall (Ranganathan and Burch, 1969; Axel, 2004; Rajiah et al., 2019). The PM itself may take a range of shapes, and both single and multiple PM bases are common (Rajiah et al., 2019). In the present study, reentry was only observed for relatively large SAC regions ( >= 8 mm radii). Although an 8 mm radius is much larger than the typical maximal PM radius of around 4.5 mm (Diaz Babio et al., 2020), the attachment between the PM base and ventricular wall often includes multiple trabeculae, which may effectively spread out the PM tugging forces across the myocardium (Ranganathan and Burch, 1969; Axel, 2004).

Furthermore, the myocardium surrounding PM insertion may be stretched either as a result of PM traction or related abnormal myocardial motion. Indeed, the region adjacent to the PMs have indeed been shown to experience increased stretch during late systole in patients with MVP, proposedly due to the prolapse of the mitral valve (Nagata et al., 2023). In future studies, clinically-measured strain profiles (Huttin et al., 2017) may be used to more accurately represent the distribution of stretch in MVP patients.

### 4.5 Effect of fibrosis on reentry

Focal fibrosis in the region of the PMs, here represented by either mesh splitting or local CV slowing in the SAC region, has been linked to PVCs and arrhythmic risk in patients with MVP (Dejgaard et al., 2018; Fulton et al., 2018; Kelley et al., 2022). Others have associated diffuse fibrosis, identified by T1-mapping *via* CMR imaging, with arrhythmic incidence in MVP patients (Chivulescu et al., 2022). Here, diffuse fibrosis was represented as global CV slowing.

We first investigated how both global and local CV slowing affected reentry inducibility and sustainability. Reducing the CV decreases the wavelength of the propagating wavefront, which is known to promote arrhythmic inducibility (Katz, 2010). On the other hand, decreasing the CV increases the activation delay between different parts of the myocardium, affecting the repolarization gradient, in turn also affecting reentry inducibility. Interestingly, reentry induction did not require the presence of fibrosis in our models, consistent with clinical findings that many MVP patients who have suffered arrhythmia or sudden cardiac death do not show evidence of fibrosis by histology or late gadolinium enhancement on CMR imaging (Muthukumar et al., 2020). Furthermore, while CV slowing generally increased the width of the vulnerability windows, a few models became non-inducible to reentry when CV was reduced (models ${M9}_{20,l}$ , ${M10}_{20,l}$ and ${M10}_{60,g}$ , Table 1). The highest ratio of sustained reentry was, however, found in the population with global CV slowing (43%) compared to local CV slowing (5%) or healthy tissue (0%), consistent with the well-known role of slowed CV in sustaining reentry (Katz, 2010).

Introducing fibrotic microstructure into our models allowed micro-reentries with highly heterogeneous activation patterns to form. These reentries could be triggered by SAC activation outside the QRS complex and T-wave, the two only vulnerable periods identified in models without microstructure. Due to the fibrotic clefts, the activation wave could move slowly through small, partly isolated portions of tissue while the remaining myocardium repolarized. The resulting reentries had a foci-like appearance, originating from the SAC region. Similar propagation patterns have been described in previous studies which modeled fibrotic microstructure using the same mesh splitting approach and which induced reentry using an electric pacing protocol (Balaban et al., 2020; Myklebust et al., 2024).

### 4.6 Limitations and future work

In this study, we simulated reentry induced by stretch in the PM insertion region. Our model was created from CMR images of an MVP patient. Due to limited image resolution challenging the reconstruction of the PMs, we did not model the PMs explicitly. Rather, we chose to focus on mechanisms of reentry within the ventricular wall. However, future studies should consider inclusion of the PMs, either *via* image-based modeling or using a simplified geometry such as a Gaussian function.

Reentry in MVP may also be induced *via* alternative mechanisms to those investigated in this study. In particular, recent clinical work on MVP patients have suggested the presence of myocardial inflammation (Miller et al., 2023), which could increase vulnerability to arrhythmia through its effect on cell membrane dynamics (Bi et al., 2022). Future *in silico* studies may investigate how pathological changes to ionic membrane currents, including accounting for inflammatory effects, could be involved in arrhythmogenesis in MVP and MAD. In addition, exercise have also been shown to trigger PVCs and nonsustained ventricular tachycardia in MVP patients (Guenancia et al., 2022). Thus, potential effects of exercise on conduction properties and membrane dynamics, including SAC time dependence, could be explored in future studies.

Previous work has shown a relationship between reentry inducibility, myocardial repolarization gradient and PVC origin (Cluitmans et al., 2021). Thus, the effect of the exact location of the PM insertion point may be addressed in future computational studies. Furthermore, PVCs of other origins than the PMs, including the outflow tract and fascicular region, have been reported in MVP patients (Sriram et al., 2013). Stretch-induced depolarization caused by PM traction may also originate from within the PM themselves rather than in the myocardium. While the origin of PVCs have frequently been mapped to the PMs in patients with MVP (Syed et al., 2016; Enriquez et al., 2019; Conti et al., 2023), the precise location is difficult to determine. Traction of the PMs may trigger depolarization anywhere between the PM insertion point and the PM tip. Such activations may give rise to reentrant circuits similar to those we observed or even micro-reentry within the PMs. Since we did not model the PM geometry, reentry occurring within the PMs was not investigated. Whether micro-reentry within the PMs can occur in MVP patients is still an open question, and the potential relation between such reentries and global arrhythmia is an area for future investigation.

Another limitation of the present study is that we chose to only include the electrophysiological effects of stretch rather than to model mechanical deformation directly. The reason for this choice is the high computational demand of simultaneously simulating cardiac mechanics and electrophysiology. Nevertheless, we aimed to look at stretch due to PM traction specifically rather than at global stretch, motivating our choice of simply simulating SAC activation in the PM insertion region.

We exclusively considered reentry induced by a single period of SAC activation. However, abnormal depolarization or repolarization caused by SAC activation may have downstream effects or accumulate across multiple cardiac cycles. Thus, the effects of PM traction across multiple sinus beats remain an area for further research.

Here, we only at looked at a general, non-specific formulation for SAC currents. However, we plan to investigate the potential effects of a more detailed SAC implementation on reentry dynamics in future studies.

We used an LDRB algorithm to assign fiber orientation in our model, and did thus not incorporate patient-specific fiber orientations. Furthermore, vortices of fiber arrangement have been observed at the PM base in experimental studies of the right ventricle (Campanale et al., 2020). The effect of varying fiber orientation at the PM base, including the influence of such fiber vortices is an interesting direction for future computational studies.

Lastly, as our goal was to investigate potential mechanisms of reentry subsequent to SAC activation, the timing of stretch of the PM implemented in this study was systematically varied rather than patient-specific. Future studies may incorporate clinical strain measurements into models of MVP in order to investigate patient-specific mechanisms of stretch and arrhythmia. The effect of other patient-specific features, such as changes in wall thickness or the PM insertion location, could also be explored *in silico*.

### 4.7 Conclusion

Stretch of the PM insertion region simulated by SAC-activation could trigger reentrant arrhythmia in a ventricular model of a patient with MVP and MAD. Fibrosis was not required to induce reentry. In models without fibrotic microstructure, we observed one of two mechanisms of reentry initiation depending on $E_{\text{SAC}}$ . For $E_{\text{SAC}}$ between −10 and −30 mV, vulnerability windows for reentry were observed during the T-wave. SAC-onset within these windows caused local depolarizations within a region of partly repolarized tissue, resulting in a functional conduction block and subsequent reentry. For $E_{\text{SAC}}$ between −40 and −70 mV, we observed vulnerability windows during the QRS complex. During this period, SAC-onset caused early repolarization in the SAC-region, which could lead to reentry of the sinus activation wave. Inclusion of fibrotic microstructure in the SAC region resulted in more heterogeneous propagation patterns and micro-reentries within this region. Furthermore, a small portion of reentries in these models were triggered outside the QRS complex or T-wave. Depending on the SAC-properties, potential fibrosis and timing of PM stretch in a patient with MVP, the above-mentioned mechanisms could be responsible for reentry in this patient group.

## Data Availability

The raw data supporting the conclusion of this article will be made available by the authors, without undue reservation.
