# Supplementary material for: Stretch of the papillary insertion triggers reentrant arrhythmia: an in silico patient study
Source: Front Physiol. 2024 Aug 19;15:1447938. doi: 10.3389/fphys.2024.1447938 (PMC11366717; doi:10.3389/fphys.2024.1447938)
Supplement: Supplementary file 4 [file DataSheet1.pdf]

Supplementary Material

1 SUPPLEMENTARY METHODS

1.1 Conductivity values

	g_{il}	g_{el}	$g_{\mathrm{it/in}}$	$g_{\mathrm{et/en}}$	$\mathrm{CV_l}$	$\mathrm{CV_{t/n}}$
Healthy CV	0.27	0.96	0.08	0.27	0.56	0.21
20% reduced CV	0.20	0.71	0.06	0.21	0.45	0.17
40% reduced CV	0.14	0.49	0.04	0.13	0.34	0.13
60% reduced CV	0.11	0.15	0.03	0.09	0.22	0.08
Purkinje layer	2.06	7.38	2.06	7.38	2.00	2.00

Table S1. Conductivity values (S/m) and conduction velocities (m/s) in the longitudinal, transverse and normal fiber direction. CVs were tuned in a 10 cm rod of myocardial tissue with a resolution equal to the average ventricular mesh resolution (530 µm). g_{il}: intracellular longitudinal conductivity. g_{el}: extracellular longitudinal conductivity. g_{it}: intracellular transverse conductivity. g_{et}: extracellular transverse conductivity. g_{in}: intracellular normal conductivity, g_{en}: extracellular normal conductivity. $\mathrm{CV_l}$: longitudinal conduction velocity. $\mathrm{CV_t}$: transverse conduction velocity. $\mathrm{CV_n}$: normal conduction velocity.

Table S2. Parameter combinations for the supplementary models. For all listed combinations, we activated SAC for a duration of 50 ms, with a maximum conductance $g_{\mathrm{SACtarget}}$ = 0.1 mS/ µF. The first column indicates the name used for referring to a type of model. Radius refers to the area with SAC activation. △ APDs describes the global APD heterogeneity by referring to the minimum and maximum APD in the tissue, resulting from the selected set of I_{Ks} scaling factors in the model. BCL refers to the duration between each sinus pacing. E_{SAC}: resting potential of the SAC current (mV). BCL: basic cycle length

Name	Radius (mm)	E_{SAC} (mV)	Tissue type	BCL (ms)	△ APDs (ms)
M22	10	-10	Healthy	500	241:290
M23	10	-10	Healthy	500	188:235
M24	10	-70	Healthy	500	241:290
M25	10	-70	Healthy	500	188:235
M26	10	-10	Healthy	1000	206:290
M27	10	-20	Healthy	1000	206:290
M28	10	-30	Healthy	1000	206:290
M29	10	-40	Healthy	1000	206:290
M30	10	-50	Healthy	1000	206:290
M31	10	-60	Healthy	1000	206:290
M32	10	-70	Healthy	1000	206:290

1.2 APD heterogeneity

In addition to the baseline APD gradient used in our main population of models, we ran simulations for two additional APD gradients in models with E_{SAC} of -10 and -70 mV. This resulted in a total of three types of APD heterogeneities, the first which is described in the Materials and Methods section.

The second type of gradient was chosen based on previous work by [1] and [2]. In accordance with these studies, we scaled the slow delayed rectifier K+ current (I_{Ks}) linearly by a factor of 1-1.5 in both the transmural and apicobasal direction. This resulted in a total scaling from 1 in the basal endocardium to 2.25 in the apical epicardium. The resulting APDs ranged from 290 ms in the basal endocardial cell to 241 ms in the apical epicardial cell.

For the third type of heterogeneity, we applied a global shortening of APDs. APD shortening reduces the wavelength for reentry and is known to affect arrhythmic vulnerability [3]. APDs were shortened by adding a factor of 1.5 to all I_{Ks} scaling factors from the second set of models, resulting in a final scaling from 2.5 in the basal endocardial cell to 5.5 in the apical epicardial cell. The APDs varied between 235 ms in the basal endocardial cell to 188 ms in the apical epicardial cell.

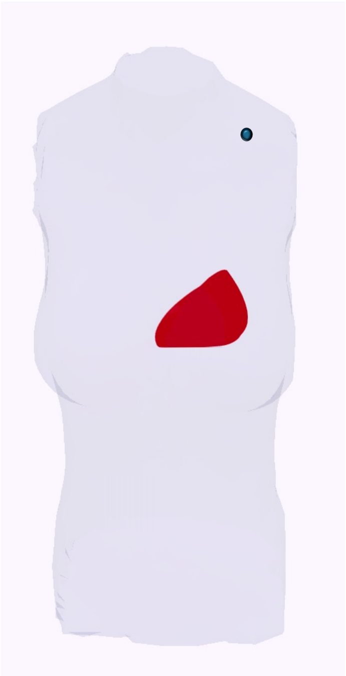

Figure S1. Visualization of the coordinate (blue point) from where we recorded the extracellular potential. The point was determined by aligning our ventricular mesh (red) with torso mesh (grey) previously developed by Uv et al. (2022) [4].

2 SUPPLEMENTARY RESULTS

2.1 Varying APD gradient

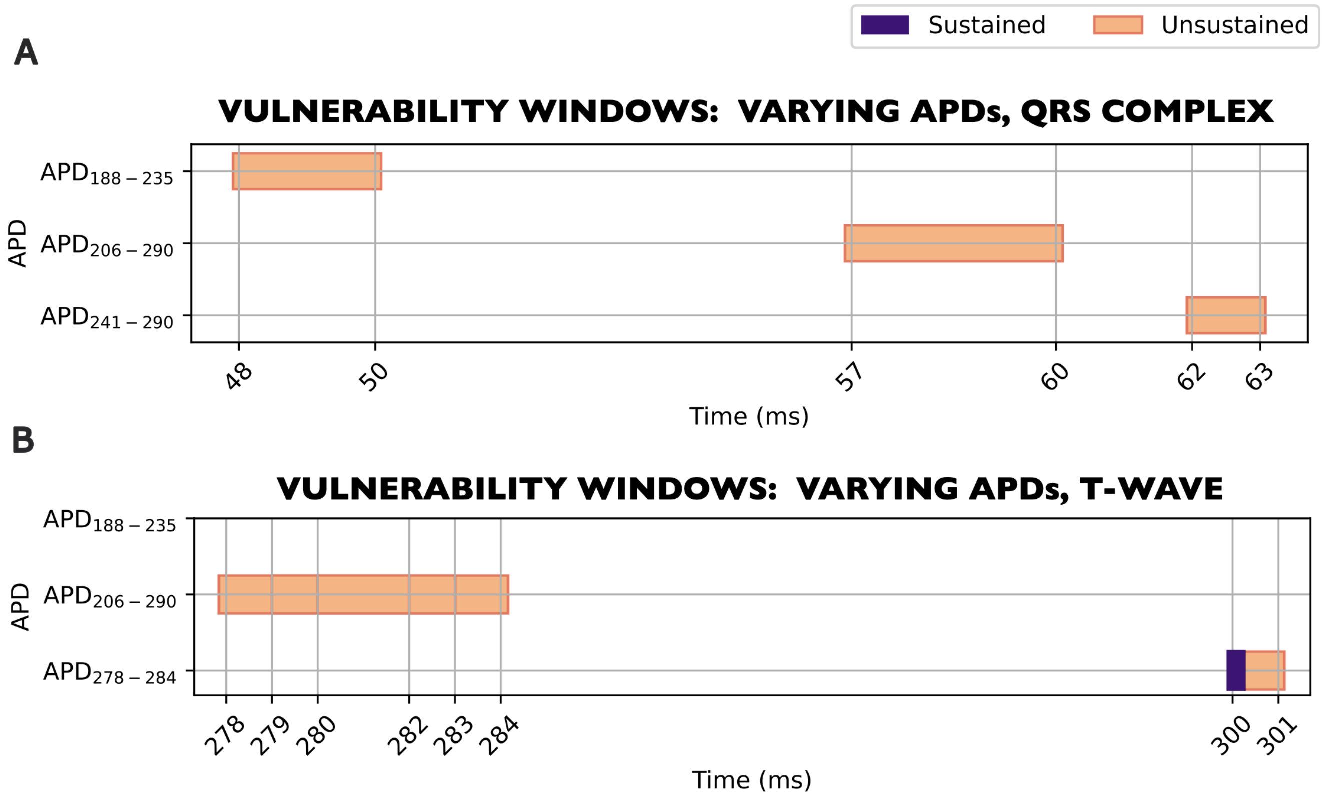

Figure S2. Grids illustrating the vulnerability to reentry for two example models with three different APD gradients. **(A)** In models with E_{SAC} = -70 mV, timings for all three vulnerability windows overlap with the QRS complex. **(B)** For E_{SAC} = -10 mV, reentry could not be induced in the model with the shortest APD gradient ($\mathrm{APD}_{188-235}$), independent of when SAC was activated. Unsustained reentries are marked orange, while the sustained reentry is marked purple.

2.2 Effect of basic cycle length

For all E_{SAC}, we explored how a change in BCL affected the reentry vulnerability windows. Figure S3 shows a vulnerability grid for models with a BCL of 1000 ms. Consistent with models with BCL of 500 ms, we observed two types of reentry: either caused by SAC-induced, early repolarization when SAC was activated during the QRS complex, or caused by SAC-induced depolarizations when SAC was activation during the T-wave. Like for BCL of 500 ms, we observed the first mechanism in models with E_{SAC} of -70 to -40 mV, and the second mechanism in models with E_{SAC} of -30 to -10 mV. However, in the population with BCL of 1000 ms, we observed one model with sustained reentry (E_{SAC} = -10 mV) and one model with no reentry (E_{SAC} = -20 mV). When changing the BCL from 500 ms to 1000 ms, the vulnerability windows were shifted to later time points. The shifts between the start of each vulnerability window were of 8, 11, 5, 7, 7 and 7 ms for E_{SAC} of -10, -30, -40, -50, -60 and -70 mV, respectively.

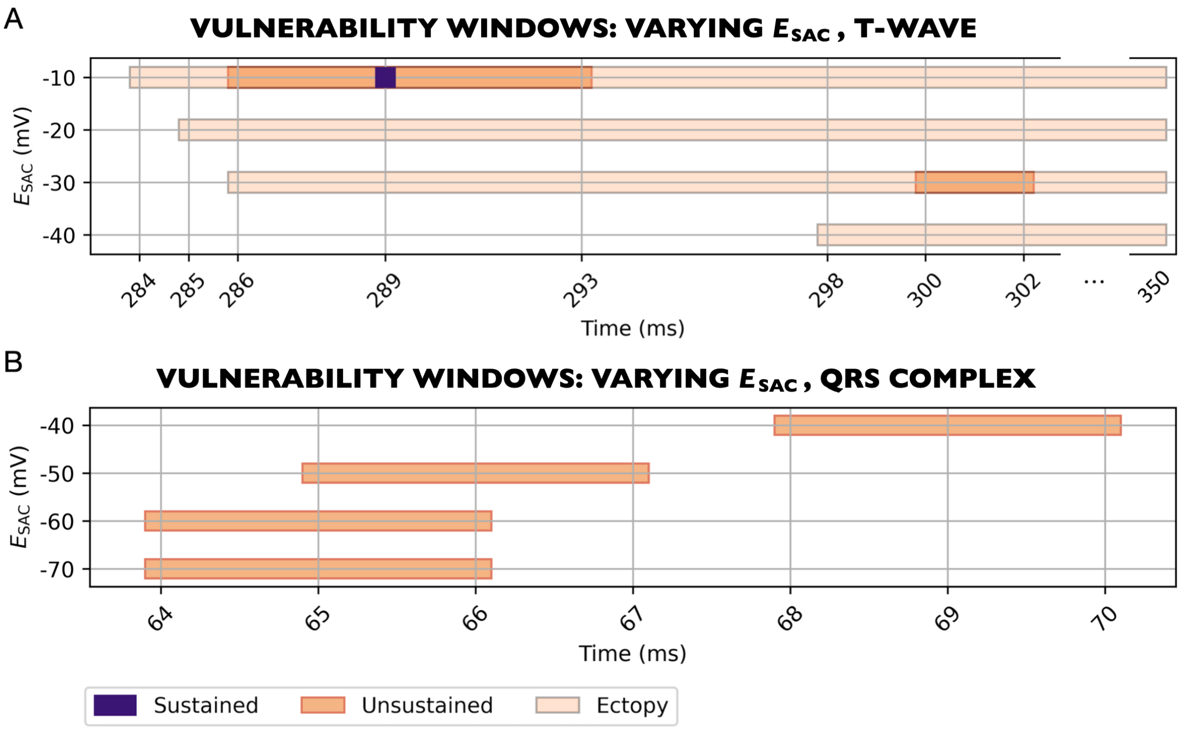

Figure S3. Vulnerability windows for reentry across all levels of E_{SAC} for models with $\mathrm{APD}_{206-290}$, 1000 ms BCL, healthy conductivities and a SAC region of 10 mm radius (models M26-M32, Table S2). The two time periods within which reentry occurred were during the T-wave (286-302, panel (**A**)) or during the QRS complex (64-70, panel (**B**)). (**A**) For E_{SAC} of -10 to -40 mV, the first time of SAC onset which triggered ectopy (light orange bar) were 284, 285, 286 and 298 ms after sinus pacing. SAC activation at any time after these points (last point measured at 350 ms) always triggered full ventricular activation. Sustained reentry is marked purple, unsustained reentry is marked dark orange. BCL: basic cycle length.

2.3 Fibrotic microstructure

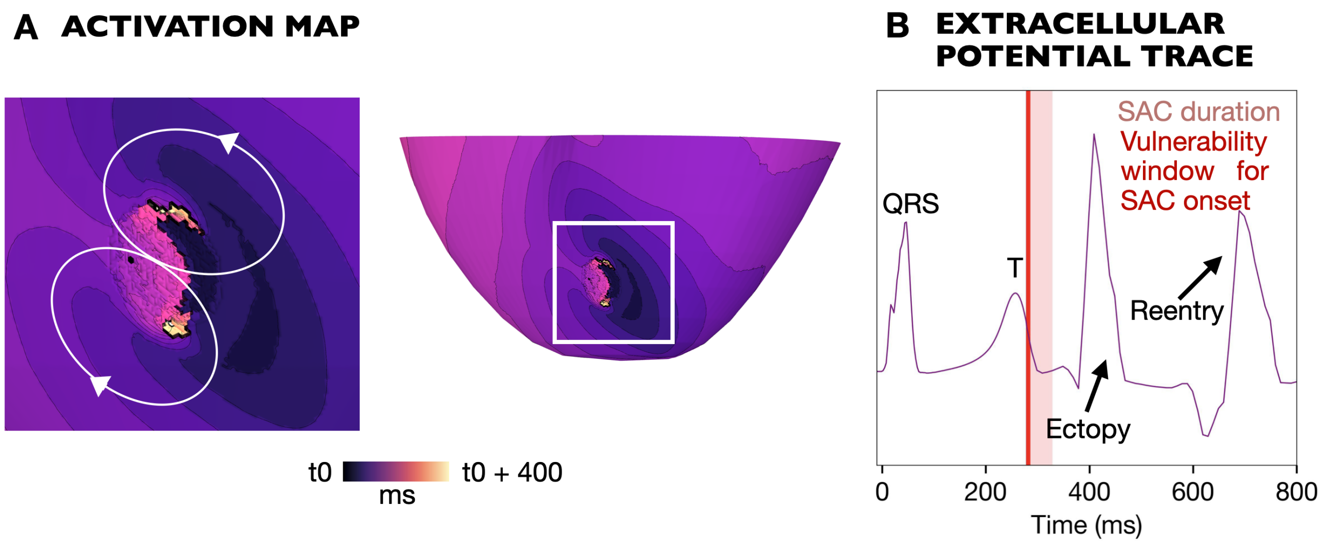

Figure S4. **(A)** Activation map for a reentry triggered by SAC-induced depolarization in a model with fibrotic microstructure in the SAC region and E_{SAC} = -10 mV. SAC was activated for 50 ms from 279 ms after the previous sinus pacing. t0 represents time of SAC onset. **(B)** Extracellular potential trace recorded from a single lead at the left shoulder.

REFERENCES

[1] Keller, D. U., Weiss, D. L., Dossel, O., and Seemann, G. (2011). Influence of I_{Ks} heterogeneities on the genesis of the t-wave: A computational evaluation. *IEEE Transactions on Biomedical Engineering* 59,311–322

[2] Sung, E., Prakosa, A., and Trayanova, N. A. (2021). Analyzing the role of repolarization gradients in post-infarct ventricular tachycardia dynamics using patient-specific computational heart models. *Frontiers in Physiology* 12, 740389

[3] Tse, G., Chan, Y. W. F., Keung, W., and Yan, B. P. (2017). Electrophysiological mechanisms of long and short qt syndromes. *IJC Heart & Vasculature* 14, 8–13

[4] Uv, J. J., Myklebust, L., Rudsari, H. K., Welle, H., and Arevalo, H. (2022). 3d simulations of fetal and maternal ventricular excitation for investigating the abdominal ecg. In *Computational Physiology: Simula Summer School 2021- Student Reports* (Springer International Publishing Cham). 13–24
